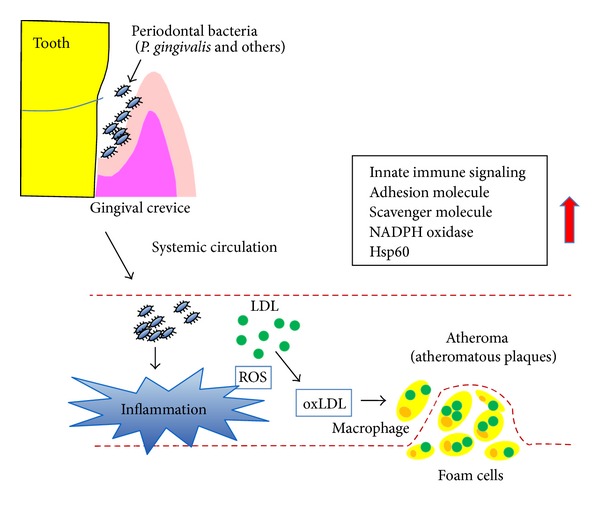# Periodontal Pathogens and Atherosclerosis: Implications of Inflammation and Oxidative Modification of LDL

**DOI:** 10.1155/2014/595981

**Published:** 2014-05-18

**Authors:** Tomoko Kurita-Ochiai, Masafumi Yamamoto

**Affiliations:** Department of Microbiology and Immunology, Nihon University School of Dentistry at Matsudo, Matsudo, Chiba 271-8587, Japan

## Abstract

Inflammation is well accepted to play a crucial role in the development of atherosclerotic lesions, and recent studies have demonstrated an association between periodontal disease and cardiovascular disease. *Porphyromonas gingivalis* and *Aggregatibacter actinomycetemcomitans*, causative agents of destructive chronic inflammation in the periodontium, can accelerate atheroma deposition in animal models. Emerging evidence suggests that vaccination against virulence factors of these pathogens and anti-inflammatory therapy may confer disease resistance. In this review, we focus on the role of inflammatory mechanisms and oxidative modification in the formation and activation of atherosclerotic plaques accelerated by *P. gingivalis* or *A. actinomycetemcomitans* in an ApoE-deficient mouse model and high-fat-diet-fed mice. Furthermore, we examine whether mucosal vaccination with a periodontal pathogen or the anti-inflammatory activity of catechins can reduce periodontal pathogen-accelerated atherosclerosis.

## 1. Introduction

Periodontitis, a chronic, destructive condition affecting a large portion of the adult population, is one of the major causes of tooth loss and characterized by a chronic infection associated with gram-negative anaerobic bacteria in the dental biofilm. It leads to irreversible destruction of tissues supporting the teeth and is clinically detectable as periodontal pockets and alveolar bone loss [1, 2]. Since periodontitis often causes bacteremia, a relationship between periodontitis and systemic diseases via periodontal pathogens has been explored [3]. Two or more periodontopathic bacteria have been detected in the cardiac valve [4, 5] and aortic aneurysm [6]; therefore, periodontal infection could also affect the progression of cardiovascular disease (CVD). However, many questions regarding this causal relationship and pathological mechanism need to be answered.

This review discusses the involvement of periodontopathic bacteria in the development of atherosclerosis. Furthermore, we mention the possibility of preventing atherosclerosis by developing a vaccine for specific bacteria or common antigens, as well as by the intake of catechin, which has both antioxidative and anti-inflammatory effects.

## 2. The Periodontal-Systemic Relationship

Epidemiological studies have suggested that periodontal infections are associated with an increased risk of CVD [7–10]. For example, it was shown that patients with periodontitis have a 19% greater risk of CVD compared to subjects without periodontitis [11, 12]. Furthermore, severe periodontitis is associated with increased intima-media thickening [13], while a systemic antibody response to a periodontal organism is associated with coronary heart disease [14]. Although observational studies suggest that such an association is independent of known confounders such as hypertension, smoking, diabetes, and obesity, this contention has not been confirmed [2]. In contrast, a recent cross-sectional study indicated that periodontitis is associated with endothelial dysfunction in a general population [15].

Considerable evidence indicates that periodontopathic bacteria may directly or indirectly contribute to cardiovascular disease, such as blood platelet aggregation, enhanced low-density cholesterol and lipoprotein deposition in the arterial walls, invasion of cardiac and carotid endothelium, and the high level of circulating- or tissue-derived inflammatory mediators [16–18]. Periodontal bacteria may directly infect atherosclerotic lesions, thus contributing to the inflammatory process. The fact that periodontopathic bacteria were detected from atheromatous plaques of coronary arteries suggested that periodontal bacteria such as* Porphyromonas gingivalis* and* Aggregatibacter actinomycetemcomitans* invade endothelial cells and induce chronic vascular inflammation [19]. Furthermore,* P. gingivalis*,* A. actinomycetemcomitans*,* Fusobacterium nucleatum*, and other oral anaerobes were shown to degrade immunoglobulin, inhibit the complement system, and produce toxic components such as lipopolysaccharide (endotoxins) and secreted molecules (exotoxins) [20, 21]. These factors sustain bacterial viability during bacteremia, which commonly occurs as a result of surgical treatments, tooth brushing, and other dental procedures [16, 22]. Chronic periodontal infections, initiated by gram-negative tooth-associated microbial biofilms, may also indirectly induce endothelial activation or dysfunction through a state of systemic inflammation as evidenced by elevated plasma acute phase proteins; proinflammatory cytokines such as interleukin-1 (IL-1), IL-6, and tumor necrosis factor-*α*; and the matrix metalloproteinase (MMP) family [23–26]. Moreover, the release of bacterial products such as outer membrane vesicles [27] or gingipains [28] from* P. gingivalis* or the release of free soluble components from* A. actinomycetemcomitans *[29] into the circulation can induce proatherogenic responses in endothelial cells. Immune activation by the pathogen-derived heat-shock protein (HSP) GroEL may also result in an autoimmune response followed by atherosclerosis via the structural similarity or “molecular mimicry” between host HSP60 and GroEL. Previous studies have shown that HSP60 is selectively located in atherosclerotic lesions rather than nonatherosclerotic areas of the arterial wall [30]. In addition, a significant correlation has been observed between anti-HSP antibody (Ab) levels and the severity of atherosclerosis. High titers of anti-HSP60 Abs have been identified in patients with carotid atherosclerosis, coronary disease, and stroke [31]. HSP of a major periodontal pathogen, such as* P. gingivalis* (GroEL), was also suggested to be a key molecule linking periodontitis (an infectious disease) with atherosclerosis (an autoimmune disease) [32, 33]. Clonal analysis of the T-cells clearly demonstrated that both human HSP60- and* P. gingivalis* GroEL-reactive T-cell populations were present in the peripheral circulation of patients with atherosclerosis [34]. In addition,* P. gingivalis* can induce platelet aggregation [35], and lipopolysaccharide (LPS) from periodontal pathogens can induce the formation of foam cells [36, 37]. Therefore, we assessed the potential of periodontal infection in atherosclerosis progression.

## 3. Role of Periodontal Infection in Atherosclerosis Progression

Animal models have been used to examine atherosclerotic pathology caused by periodontopathic bacteria.* P. gingivalis* has been reported by our and other groups to accelerate atherosclerosis in ApoE-KO or apolipoprotein E-deficient spontaneously hyperlipidemic (ApoE^shl^) mice [38–41].* A. actinomycetemcomitans* bacteremia also aggravated atherosclerosis in ApoE^shl^ mice [42, 43]. In addition to murine models, rabbits with induced periodontal disease accumulated fatty streaks in the aorta earlier than periodontally healthy animals [44].* P. gingivalis* bacteremia also enhanced atherosclerosis in normocholesterolemic and hypercholesteromic pigs [45]. Thus, in addition to the detection of live periodontal pathogens in human atherosclerotic plaques,* in vivo* experiments in a variety of animal models have corroborated that* P. gingivalis* can enhance atherogenesis.

## 4. Endothelial Cell Activation

Periodontal bacteria and their products can initiate an inflammatory response in the periodontal tissues with systemic consequences. Moreover, bacteria and their products can gain access to the circulation during dental procedures such as scaling and root planning, or even after vigorous home care, especially in patients with periodontitis. Once in the blood stream, bacterial antigens can induce a systemic immune response. Periodontal bacteria in the blood can be carried to distant sites such as the heart tissues freely in the circulation or within circulating cells such as monocytes, neutrophils, or platelets and can initiate pathogenic processes. Tissue or cellular invasion is a key virulence property of many bacterial species. Periodontal pathogens including* P. gingivalis* also adhere to and invade various human vascular cells in culture and animal model [40, 46, 47]. Furthermore,* P. gingivalis* can simulate monocyte adhesion to human umbilical vein endothelial cells [40]. Endothelial activation and increased expression of adhesion molecules and chemokines comprise an initial step in the development of atherosclerotic lesions.* A. actinomycetemcomitans *infection of ApoE^shl^ mice resulted in increased expression of intercellular adhesion molecule 1 (ICAM-1), monocyte chemoattractant protein 1 (MCP-1), E-selectin, P-selection, chemokine (C-C motif) ligand 19 (CCL19), CCL21, and C-C chemokine receptor 7 in the aorta [42]. Coculture of human aortic endothelial cells with* P. gingivalis* also increased the expression of ICAM-1, vascular cell adhesion molecule 1 (VCAM-1), MCP-1, and E-selectin by a fimbriae-dependent activation mechanism [48, 49]. Chemical inhibition of endocytosis blocked the upregulation of MCP-1, indicating the need of bacterial invasion for MCP-1 stimulation [50]. In contrast, stimulation of endothelial cells with LPS, an outer membrane protein, and HSP60 derived from* P. gingivalis *had only a slight stimulatory effect on ICAM-1 and VCAM-1 expression [51].

## 5. Toll-Like Receptor- and Nod-Like Receptor-Mediated Responses

Exposure of periodontal pathogens to endothelial cells may result in increased expression and interaction with Toll-like receptors (TLRs).* A. actinomycetemcomitans *infection of ApoE^shl^ mice enhanced mRNA expression of TLR2, TLR4, TLR-9, and nucleotide binding oligomerization domain 1 (NOD-1) in the aorta [42, 43]. Treatment with the heat-killed (H.K.)* A. actinomycetemcomitans* or* A. actinomycetemcomitans* LPS also increased TLR4, NOD1, and NOD2 expression. However, the order of atherosclerosis extent was as follows: live* A. actinomycetemcomitans* > H.K.* A. actinomycetemcomitans* >* A. actinomycetemcomitans* LPS. Furthermore, a significant difference in atherosclerotic lesion size was observed between* A. actinomycetemcomitans*-challenged mice and* A. actinomycetemcomitans* LPS-challenged mice. ApoE knockout mice challenged with* P. gingivalis* also expressed TLR2 and TLR4 in aortic tissue [39], while challenge with an invasion-impaired* P. gingivalis* fimbriae-deficient mutant did not upregulate TLRs, suggesting that innate immune recognition of invasive bacteria is a prerequisite for the acceleration of atherosclerosis. In contrast, ApoE(–/–) and TLR4(–/–) double-knockout mice were markedly more susceptible to atherosclerosis after oral infection with* P. gingivalis*, demonstrating an atheroprotective role for TLR4 in response to* P. gingivalis* infection [52].

## 6. Oxidative Stress-Mediated Mechanisms

Lipid peroxidation plays an important role in many diseases [53]. In particular, low-density lipoprotein (LDL) oxidation may be a key step in the development of atherosclerosis [54]. Oxidation of LDL is essential for its accumulation within the macrophages and for the formation of foam cells, which can upregulate proatherogenic chemokines and adhesion molecules [55] and induce interleukin 6 (IL-6), tumor necrosis factor alpha, and C-reactive protein secretion [56].* A. actinomycetemcomitans *infection of ApoE^shl^ mice promoted LDL oxidation, as indicated by the marked upregulation of 4-hydroxynoneal, oxidated LDL, and phospholipase A2 in the aorta, ox-LDL, 8-oxo-2′-deoxyguanosine, and myeloperoxidase serum levels, and aortic expression of nicotinamide adenine dinucleotide phosphate oxidase, caveolin-1, and RAGE [43].* P. gingivalis* also increased oxidative modification of LDL [57, 58] and rupture of atherosclerotic plaque through induction of MMP [59]. Indeed, patients with periodontitis have increased levels of lipid peroxidation in plasma, saliva, and gingival crevicular fluid [60], and these levels have been correlated with the severity of periodontal disease [60, 61]. Coincubation of a murine macrophage cell line with* P. gingivalis* in the presence of LDL resulted in the formation of foam cells in a dose-dependent manner [62].

## 7. Hyperlipidemia-Induced Atherosclerosis

CVD remains the leading cause of morbidity and mortality in the Western world. A sedentary lifestyle and Western dietary habits may contribute to this increased risk of CVD development [63, 64]. For example, consumption of a diet rich in saturated fat is positively associated with elevated plasma lipid levels and a state of subacute chronic inflammation, which are important risk factors promoting both the onset and development of CVD [65, 66]. Research on a correlation between periodontopathic bacteria and atherosclerosis has been performed using hyperlipidemic animals [38, 39, 45, 67]. Furthermore, a previous study demonstrated that an increase in atherosclerosis is associated with* P. gingivalis* infection in B6.ApoE^shl^ mice, but not in wild-type mice fed a regular chow diet (RD) [68]. In contrast,* P. gingivalis* injection significantly increased the size and lipid content of atherosclerotic lesions in C57BL/6 mice fed a high fat diet (HFD) [69]. Inoculation of* C. pneumoniae* into C57BL/6 mice fed a HFD also accelerated hypercholesterolemia-induced atherosclerosis [70]. In contrast, chlamydial inoculation into C57BL/6 mice fed the RD did not affect aortic lipid accumulation, although inflammatory changes were induced [71]. Upon infection with* P. gingivalis*, gene expression profiles of the aorta and liver in wild-type mice showed proatherogenic profiles, even in animals fed the RD [68]. Challenge with* P. gingivalis* also induced inflammation associated with elevated serum IL-6, IL-8, and MCP-1 levels, even in C57BL/6 mice fed the RD; however, plaque formation was only slightly elevated. Therefore, the inflammatory response caused by periodontal infection may act in concert with hyperlipidemia to exacerbate atherosclerotic lesion formation.

## 8. Prevention of Atherosclerosis by Mucosal Vaccination

Since several possible mechanisms may be involved in the acceleration of atherosclerosis by periodontal pathogens, the prevention of periodontal infection may be an effective way to reduce the induction of atherosclerosis, as well as periodontitis. Thus, the prevention of periodontitis might be relevant not only for oral, but also for systemic health. Atherosclerosis and inflammation in ApoE^shl^ and HFD-fed C57/BL6 mice challenged with* P. gingivalis* were effectively prevented by nasal immunization with the 40 kDa outer membrane protein of* P. gingivalis* [41, 69]. Furthermore, since HSP60 (GroEL) from* P. gingivalis* can trigger molecules linking infectious periodontitis and autoimmune atherosclerosis [32, 33], mucosal administration of a relevant autoantigen is an effective method of attenuating autoimmune disease by inducing an unresponsive state of tolerance [72, 73]. Sublingual immunization with* P. gingivalis* GroEL controlled the acceleration of disease by* P. gingivalis* infection with an increase in the GroEL antibody [74]. Therefore, mucosal vaccination with GroEL may control inflammation and the progression of atherosclerosis due to periodontopathic bacterial infection.

## 9. Prevention of Atherosclerosis Using an Anti-Inflammatory Agent

Previous studies have indicated that early activation of inflammatory mediators in response to a challenge with periodontal pathogens may be associated with periodontitis-associated atherosclerosis [41, 42, 69]. Therefore, oral inflammatory diseases and systemic inflammation caused by periodontal infection may contribute to atherosclerosis. Green tea is a popular drink worldwide, and consumption of green tea has been suggested to prevent the development of a variety of diseases, including diabetes, hypertension, cancer, and cardiovascular diseases [75]. The effects of green tea are attributed to its abundant and biologically active ingredient catechin, or epigallocatechin-3-gallate (EGCG), which has antioxidative [76], anti-inflammatory [77], and antiangiogenic [78] effects. Consumption of EGCG in ApoE knockout mice decreased atherosclerotic lesions, proinflammatory cytokines, and inflammatory- and oxidative stress-related mediators in the serum and aorta induced by* P. gingivalis* [79]. Therefore, previous catechin consumption may be useful for the prevention of pathogen-accelerated atherosclerosis.

## 10. Conclusions

The association between periodontal disease and atherosclerotic cardiovascular disease is supported by a large body of evidence. Periodontal bacteria were shown to accelerate the progression of atherosclerosis in ApoE knockout mice, rabbits, and pigs. Furthermore, observations have suggested that inflammation caused by periodontopathic bacteria may play a synergistic role with other preexisting factors, such as hyperlipidemia, resulting in the development of atherosclerosis. These findings support the hypothesis that a periodontal pathogen is not an independent risk factor but instead acts in concert with hyperlipidemia to exacerbate atherosclerosis lesion formation. However, whether live bacteria are necessary for this process, or if its component alone is sufficient to cause an increase in atherosclerotic damage, remains unclear. In a complex tissue, such as an atherosclerotic lesion, innate signals can originate from several sources and promote atherogenesis through an association with pattern-recognition receptors (PRRs). These signals include various extracellular activation cascades and intracellular signaling pathways and lead to effective clearance of infectious agents and induction of inflammatory responses. Since live bacteria can activate multiple PRRs, they may induce a more significant inflammatory response than their components. Finally, the best method to prevent atherosclerosis is by blocking bacterial invasion and bacteria-induced inflammation and lipid peroxidation. Previous results have demonstrated that atherosclerosis and inflammation with lipid peroxidation are accelerated in ApoE-deficient mice after an infection of periodontal bacteria (Figure 1) and that these can be prevented by mucosal immunization with bacterial products or an anti-inflammatory agent.

## Figures and Tables

**Figure 1:**